# Perfusion vs non-perfusion computed tomography imaging in the late window of emergent large vessel ischemic stroke: A systematic review and meta-analysis

**DOI:** 10.1371/journal.pone.0294127

**Published:** 2024-01-02

**Authors:** Jose Danilo B. Diestro, Abdelsimar T. Omar, Yu-qing Zhang, Teruko Kishibe, Alexander Mastrolonardo, Melissa Mary Lannon, Katrina Ignacio, Eduardo Pimenta Ribeiro Pontes Almeida, Anahita Malvea, Ange Diouf, Arjun Vishnu Sharma, Qingwu Yang, Zhongming Qiu, Mohammed A. Almekhlafi, Thanh N. Nguyen, Atif Zafar, Vitor Mendes Pereira, Julian Spears, Thomas R. Marotta, Forough Farrokhyar, Sunjay Sharma

**Affiliations:** 1 Department of Health Research Methods, Evidence, and Impact, McMaster University, Hamilton, Ontario, Canada; 2 Division of Diagnostic and Therapeutic Neuroradiology, Department of Medical Imaging, Unity Health- St Michael’s Hospital, University of Toronto, Toronto, Ontario, Canada; 3 Division of Neurosurgery, Department of Surgery, McMaster University, Hamilton, Ontario, Canada; 4 CEBIM (Center for Evidence Based Integrative Medicine)-Clarity Collaboration, Guang’anmen Hospital, China Academy of Chinese Medical Sciences, Beijing, China; 5 Nottingham Ningbo GRADE Center, The University of Nottingham Ningbo, Ningbo, China; 6 Health Sciences Library, St. Michael’s Hospital, Unity Health Toronto, Toronto, Ontario, Canada; 7 Li Ka Shing Knowledge Institute, St. Michael’s Hospital, Unity Health Toronto, Toronto, Ontario, Canada; 8 Michael G. DeGroote School of Medicine, McMaster University, Hamilton, ON, Canada; 9 Department of Clinical Neurosciences, Cumming School of Medicine, University of Calgary, Alberta, Canada; 10 University Health Network—Toronto General Hospital, Toronto, Ontario, Canada; 11 Division of Neurosurgery, Department of Surgery, Unity Health- St Michael’s Hospital, University of Toronto, Toronto, Ontario, Canada; 12 Department of Neurology and Critical Care, McMaster University, Hamilton, ON, Canada; 13 Department of Neurology, Xinqiao Hospital and The Second Affiliated Hospital, Army Medical University (Third Military Medical University), Shapingba District, Chongqing, China; 14 Department of Neurology, The 903rd Hospital of The People’s Liberation Army, Xihu District, Hangzhou, China; 15 Department of Clinical Neurosciences, Radiology, and Community Health Sciences, Cumming School of Medicine at the University of Calgary, Calgary, Alberta, Canada; 16 Hotchkiss Brain Institute and O’Brien Institute for Public Health, Cumming School of Medicine at the University of Calgary, Calgary, Alberta, Canada; 17 Department of Neurology, Boston University School of Medicine, Boston, Massachusetts, United States of America; 18 Department of Radiology, Boston Medical Center, Boston University School of Medicine, Boston, Massachusetts, United States of America; 19 Department of Medicine, Division of Neurology, Unity Health- St. Michael’s Hospital, University of Toronto, Toronto, Ontario; 20 Department of Global Health, McMaster University, Hamilton, Ontario, Canada; 21 Department of Surgery, McMaster University, Hamilton, Ontario, Canada; Foshan Sanshui District People’s Hospital, CHINA

## Abstract

**Background:**

Guidelines recommend the treatment of emergent large vessel ischemic stroke (ELVIS) patients presenting beyond 6 hours of last known well time with endovascular thrombectomy (EVT) based on perfusion computed tomography (CT) neuroimaging. We compared the outcomes (long-term good clinical outcomes, symptomatic intracranial hemorrhage (sICH), and mortality) of ELVIS patients according to the type of CT neuroimaging they underwent.

**Methods:**

We searched the following databases: Medline, Embase, CENTRAL, and Scopus from January 1, 2015, to June 14, 2023. We included studies of late-presenting ELVIS patients undergoing EVT that had with data for non-perfusion and perfusion CT neuroimaging. We followed the Preferred Reporting Items for Systematic Reviews and Meta-analyses guidelines. Data were pooled using a random effects model.

**Results:**

We found 7 observational cohorts. Non-perfusion versus perfusion CT was not statistically significantly different for both long-term clinical (n = 3,224; RR: 0.96; 95% CI 0.86 to 1.06; I^2^ = 18%) and sICH (n = 3,724; RR: 1.08 95% CI 0.60 to 1.94; I^2^ = 76%). Perfusion CT had less mortality (n = 3874; RR: 1.22; 95% CI 1.07 to 1.40; I^2^ = 0%). The certainty of these findings is very low because of limitations in the risk of bias, indirectness, and imprecision domains of the Grading of Recommendations, Assessment, Development and Evaluations.

**Conclusion:**

The use of either non-perfusion or perfusion CT neuroimaging may have little to no effect on long-term clinical outcomes and sICH for late-presenting EVT patients. Perfusion CT neuroimaging may be associated with a reduced the risk of mortality. Evidence uncertainty warrants randomized trial data.

## Introduction

The treatment of emergent large vessel occlusion ischemic stroke (ELVIS) has been revolutionized by endovascular thrombectomy (EVT) [1]. The current American Heart Association Guidelines recommend the treatment of ELVIS presenting within 6 hours of symptom onset or last known well time with EVT on the basis of clinical status and non-perfusion neuroimaging, consisting of a non-contrast computed tomography (NCCT) scan and a CT angiogram (CTA). Beyond the 6-hour window the AHA guidelines recommend (Grade 1A) the use of advanced imaging such as automated perfusion imaging to quantify degree of mismatch and ischemic cores size before deciding on performing EVT [2, 3]. This recommendation is based on randomized controlled trials, DEFUSE 3 and DAWN, that demonstrated EVT benefit beyond 6 hours [4, 5]. DEFUSE 3 utilized narrow inclusion criteria based on values obtained via automated perfusion imaging (RAPID, iSchemaView, Menlo Park, CA). Other more recent guidelines have not been as restrictive in their imaging paradigm recommendation [6].

The use of automated perfusion scanning aims to identify patients who already have large ischemic cores. It is hypothesized that EVT in these patients may either be futile or pose an increased risk of hemorrhagic transformation. A recent large retrospective observational study, CT for Late EndovasculAr Reperfusion (CLEAR), found that ELVIS patient undergoing EVT utilizing non-perfusion CT neuroimaging for decision-making had comparable clinical outcomes compared to those that utilized advanced neuroimaging with CT perfusion or magnetic resonance imaging [7].

Access to the required perfusion CT neuroimaging may be difficult for smaller centers, primary stroke centers and those in low- or middle-income countries. Thus, strict adherence to the AHA guidelines may even result in ELVIS patients being denied EVT in centers without CT perfusion. We conducted a systematic review and meta-analysis to determine if the use of non-perfusion CT neuroimaging (non-contrast computed tomography scan and CTA) differs from that of perfusion CT neuroimaging (non-contrast computed tomography scan, CTA and perfusion scan) in terms of long-term clinical outcomes (modified Rankin scale of 0–2 at 90 days or more), symptomatic intracranial hemorrhage (sICH) and mortality for ELVIS patients undergoing EVT in the late window (more than 6 hours after last time seen well).

## Materials and methods

### Design

This systematic review was conducted using a predefined protocol was registered with PROSPERO (CRD42022375635: https://www.crd.york.ac.uk/prospero/display_record.php?ID=CRD42022375635). The PRISMA (Preferred Reporting Items for Systematic Reviews and Meta-Analysis) statement was used to ensure rigorous methodology and high-quality reporting. The PRISMA statement consists of a flow diagram and a checklist.

### Search strategy

An information specialist (TK) worked with the lead authors (JBD, ATO) to develop the search strategy. We conducted the search on Medline (Ovid), Embase (Ovid), the Cochrane Central Register of Controlled Trials (Ovid) and Scopus (Elsevier). Keywords and Medical Subject Heading (MeSH) terms related to our research question were used. The search strategy and search terms for each database is detailed in the (S1 Appendix).

### Study selection

We included cohort studies and clinical trials published between January 1, 2015 to June 14, 2023 on EVT for ELVIS with data on at least one of our outcomes, mRS at 90 days or more, symptomatic intracranial hemorrhage and mortality. We included studies with adult (≥ 18 years old) acute ischemic stroke patients presenting in the delayed time window (more than 6 hours after stroke onset or last known well time) who underwent either non-perfusion or perfusion CT neuroimaging as described above prior to undergoing EVT for ELVIS. We included all publications without language restrictions. We excluded reviews, letters to the editors, editorials, conference articles, and studies with a sample size of less than 20 participants.

Only patients in the late window were included as perfusion CT neuroimaging is not recommended for early window patients [8]. We limited our search to studies published after 2015 because the successful landmark studies for EVT for ELVIS were published in this year. Including older studies would have risked having studies with older generation thrombectomy devices. We did not include MRI based studies as we limited our review to CT based imaging modalities only. Lastly, we communicated with authors of studies with possible overlapping patients. Possible overlaps were identified by meticulously going through the list of authors and the institutions involved as detailed in the study supplements. If overlapping patients were confirmed, we excluded either the smaller study with duplicated data or only the replicated patients if the corresponding authors gave access to the raw data.

### Interventions

The current standard of care for stroke patients presenting in the late window is perfusion CT neuroimaging that includes, CT perfusion, NCCT and CTA; thus, we considered this to be our comparator. We defined non-perfusion CT neuroimaging, NCCT and CTA only, as the intervention. Non-perfusion CT neuroimaging included patients who underwent both single phase and multiphase collateral imaging. Perfusion CT neuroimaging included all those who underwent perfusion imaging even if a standard automated software was not used.

### Screening and data abstraction

The final list of studies for consideration were uploaded into the Covidence, a web-based collaboration software platform [9]. Two authors from the team screened all titles with abstracts and full texts prior to inclusion or exclusion. In cases of disagreements, the study lead (JBD) resolved conflicting decisions. All studies for full text screening were reviewed by two authors, JBD and another author. Discrepancies were resolved by a third author (ATO). The references of studies that were related to our topic but were not included in the final list of studies were also hand searched for possible pertinent articles.

Study characteristics including title, lead author, country, study design, study duration, study funding, population description, inclusion and exclusion criteria, participant demographics, stroke time metrics and the outcomes of interest were independently extracted by the lead author (JBD) and another author (AVS). Discrepancies were resolved by a third author (ATO).

### Outcomes

The primary outcome of the study is good clinical outcomes at 90 days and beyond after the stroke. Good clinical outcome was defined as a modified Rankin scale (mRS) score of 0–2 (no symptoms to slight disability) [10]. The secondary outcomes two outcomes were symptomatic intracranial hemorrhage (sICH) and mortality. There were many definitions of sICH [11]. To capture all important safety events, we included all patients flagged as sICH according to the definitions used by the individual studies. These three outcomes are standard across randomized controlled trials evaluating the safety and efficacy of EVT for ELVIS [1]. Risk ratios were selected as the preferred measure to convey the effect size, as they offer a relatively more intuitive representation compared to odds ratios for health professionals [12].

### Quality assessment

The risk of bias was assessed using the ROBINS-I (The Risk Of Bias In Non-randomized Studies–of Interventions assessment tool) for each eligible study by 2 reviewers (JBD and ATO). Conflicts were resolved by discussion. The tool evaluates observational studies based on the following bias domains: (1) bias due to confounding, (2) bias in selection of participants into the study, (3) bias in the classification of interventions, (4) bias due to deviations from intended interventions, (5) bias due to missing data, (6) bias in measurement of outcomes, and (7) bias in selection of the reported result. Risk of bias judgments for each domain were then classified [13]. An overall risk of bias judgment was formed for the entire study and particular outcome: low risk of bias (low risk for all domains), moderate risk of bias (low or moderate risk for all domains), serious risk of bias (serious risk in at least one domain but not at critical risk for any domain), critical risk of bias (critical risk of bias in at least one domain) and no information (not at serious or critical risk of bias and there is a lack of information in one or more key domains) [14].

### Subgroup and sensitivity analyses

We planned to perform subgroup analyses based on study design (randomized trials versus observational cohorts), last known well time and date of publication (before and after 2018).

### Statistical analysis

We conducted a meta-analysis of binary outcomes–reported as relative risks with 95% confidence intervals–for good functional outcome, sICH, and mortality. We used R 4.2.2 (R Foundation for Statistical Computing, Vienna, Austria) on the RStudio platform and the meta and metafor packages for analysis and data visualization [15, 16]. The data were synthesized using a random-effects model using the Mantel-Haenszel method and the Paule-Mandel estimator. The Paule-Mandel method to estimate the variance of the true distribution of effect sizes or τ^2^ was found to be more robust for binary data than the DerSimonian-Laird estimator, which can be biased particularly in reviews with high heterogeneity or a small number of studies [17, 18]. Statistical heterogeneity was detected using the χ^2^ test and the degree of heterogeneity was quantified using the I^2^ statistic, classified into low (0–40%), moderate (41–60%), and high (>60%). We evaluated for possible publication bias visually by constructing funnel plots. We also quantitatively assessed funnel asymmetry using Egger’s test, with a significant p value set at <0.05.

### Certainty of evidence

The GRADE approach was used to evaluate the certainty of evidence. The GRADE system offers a transparent and structured approach to developing evidence summaries and recommendations in healthcare, providing a comprehensive framework for guideline developers regardless of evidence quality, while acknowledging the need for judgments despite its systematic methodology [19]. We assessed the whole body of evidence according to the following domains: risk of bias, inconsistency, indirectness, imprecision and publication bias. We set our appreciable harm/ benefit rate at 25% for good long term clinical outcomes [20]. We set it lower at 10% for both sICH and mortality as these are safety outcomes and smaller differences are more important to detect. Results and concluding statements were worded according to the GRADE guidelines [21]. The guidelines necessitated that informative statements to communicate the findings of systematic reviews be based on the level of certainty of the evidence (high, moderate, low and very low) and the size of the effect (large, moderate, small and trivial).

## Results

### Search results

**Fig 1:**
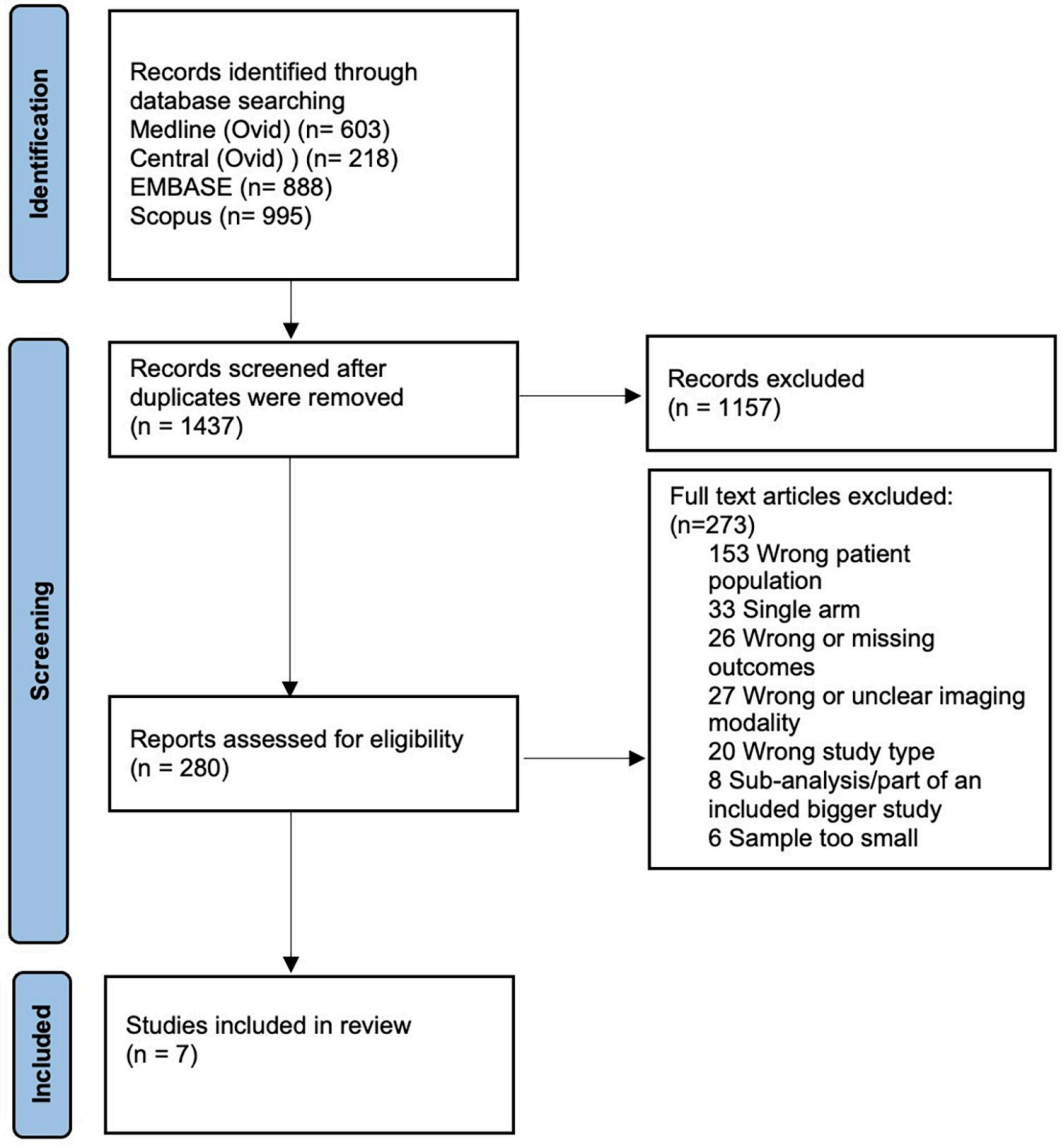
PRISMA flow diagram of study selection.

The PRISMA flow diagram is presented in Fig 1. After searching the four databases and removing duplicates, we had 1437 records. Of these 1157 were irrelevant based on title and abstract screening. After screening the full text of 280 articles we found 7 observational studies fulfilling our inclusion criteria.

### Characteristics of excluded studies

We found a paper by Nogueira et al. [22], that initially made our final list of patients. However after emailing the authors, we found this study entirely duplicated in the larger multicenter cohort by Nguyen et al. [7]. Thus, we excluded this paper. We also found another 25 studies that met all the inclusion criteria for our review except for having both an intervention and control arms (S1 Table).

### Characteristics of included studies

We included 7 observational studies in our review (Table 1). No randomized clinical trials specific to our research question was found. Ninety-day outcomes (good clinical outcomes and mortality) were available for all but two of the studies. Dhillon et al. used a longer follow-up at 180 days for good long term clinical outcomes and in-hospital mortality. Alsahli et al. did not report data for symptomatic intracranial hemorrhage and mortality. Multi-center cohorts were inspected for common centers. Communication with the Lausanne group confirmed duplicate contributions to both multicenter cohorts in our study [7, 23]. We obtained the pooled data excluding Lausanne’s data from the corresponding author. One study obtained data from two randomized trials that utilized cranial MRIs [24]. After correspondence with the authors, we were able to obtain data that excluded patients with MRIs.

**Table 1:** Included studies.

Lead Author, Publication Year	Study design: Country	Total number of participants for primary outcome Non-perfusion/ Perfusion	Age: Median/mean (IQR/SD) Non-perfusion/ Perfusion	Baseline NIHSS: Median (IQR) Non-perfusion/ Perfusion	Sex: Female n (%)Non-perfusion/ Perfusion	Given IV rTPA n (%)Non-perfusion/ Perfusion	Clot location: n (%) for each, M1, M2, Carotid Termination, Tandem: Non-perfusion	Clot location: n (%) for each, M1, M2, Carotid Termination, Tandem: Perfusion	Successful Thrombectomy outcome: TICI 2B-3 n (%)Non-perfusion/ Perfusion	Main study findings
Miao 2023 [24]	Post-hoc RCT analysis; China	534: 222/312	68 (56–74)/ 65 (55–72)	15 (11–19)/ 15 (10–19)	86 (38.7)/ 105 (33.7)	6 (2.7)/ 9 (2.9)	M1: 365 (68.4) M2: 81 (15.2) ICA: 88 (16.5)	M1: 143 (64.4) M2: 33 (14.9) ICA: 46 (20.7)	Not reported	No significant difference in all three outcomes in both early and late windows.
Porto 2022 [43]	Multicenter observational cohort: International collaboration with USA based lead author	699: 419/280	68 (57–78)/ 70 (60–81)	15 (10–19)/ 14 (8–19)	226 (53.9)/ 146 (52.1)	59 (14.1)/ 40 (14.3)	M1: 178 (42.5) M2: 101 (24.1) ICA: 140 (33.4)	M1: 137 (48.9) M2: 46 (16.4) ICA: 97 (34.6)	351 (84.2)/ 246 (89.1)	No significant difference in all three outcomes.
Almekhlafi 2022 [23]	Multicenter Observational cohort: International collaboration with Canada based lead author	773: 294/479	70(20)/ 70(22)	15(11)/ 16(10)	122(53.3)/ 185(48.8)	19(8.3)/ 36(9.5)	M1 144(62.9) M2 19 (8.3) ICA: 49 (21.4) Tandem: 36 (15.7)	M1 223(58.8) M2 63(16.6) ICA: 87 (23) Tandem: 76 (20.1)	199 (86.9)/ 298(77.8)	Basic CT neuroimaging had more sICH. No significant difference for both long term outcomes and mortality.
Nguyen 2022 [7]	Multicenter observational cohort: International collaboration with US lead author	1839: 754/1085	71 (58–81)/ 69 (58–80)	17 (13–21)/ 16 (11–19)	273 (51.1)/ 406 (54.0)	126 (23.6)/ 91 (12.1)	M1: 300 (56.2) M2: 73 (13.7) ICA: 161 (30.2)	M1: 430 (57.2) M2: 160 (21.3) ICA: 162 (21.5)	474 (88.9)/ 670 (89.5)	No significant difference in all three outcomes.
Dhillon 2022 [25]	Observational cohort: UK	508: 319/189	Not reported	Not reported	Not reported	Not reported	Not reported	Not reported	523 (78.3)/ 318 (84.1)	Main outcome of study was discharge MRS. Favored advanced CT neuroimaging. No significant difference in our 3 outcomes.
Dekker 2021 [27]	Observational cohort: Netherlands	149: 119/30	67.4 (14.7)/ 65.8 (14.4)	9 (7–10)/ 7 (5–9)	48 (56.5)/ 11 (52.4)	20 (23.8)/ 4 (19)	M1: 47 (58) M2: 10 (12.3) ICA: 24 (29.6)	M1: 10 (47.6) M2: 5 (23.8) ICA: 5 (23.8) Other: 1 (4.8)	45 (54.2)/ 13 (68.4)	More hemorrhage was seen in the advanced CT neuroimaging arm. No significant difference for long term outcomes and mortality.
Alsahli 2019 [26]	Observational cohort: Australia	91: 56/35	Not reported	Not reported	Not reported	Not reported	Not reported	Not reported	Not reported	Advanced CT neuroimaging with more favorable long term outcomes. No data for sICH and mortality.

* MCA, Middle cerebral artery; M1, First segment of the MCA; M2, Second segment of the MCA; ICA, Internal Carotid Artery; SD, standard deviation; IQR, interquartile range; TICI, Thrombolysis in cerebral infarction; CT, compute tomography; MRI, magnetic resonance imaging; sICH, symptomatic intracranial hemorrhage; IV tpa, Intravenous recombinant tissue plasminogen activator

*All data are based on the full cohorts as published.

### Quality assessment

After assessing all the included studies for all three outcomes of interest, long term clinical outcomes, sICH and mortality, with the comprehensive ROBINS-I tool, we found all the included studies to have serious risk of bias for all the outcomes (S2 Appendix). This is because of the lack of uniform adjustment for confounders for all studies. While some of the studies adjusted for confounders [7, 23, 25], the covariates adjusted for and the adjustment methods were not consistent across the studies. Consequently, we made the decision to use the unadjusted data.

### Outcomes

**Fig 2:**
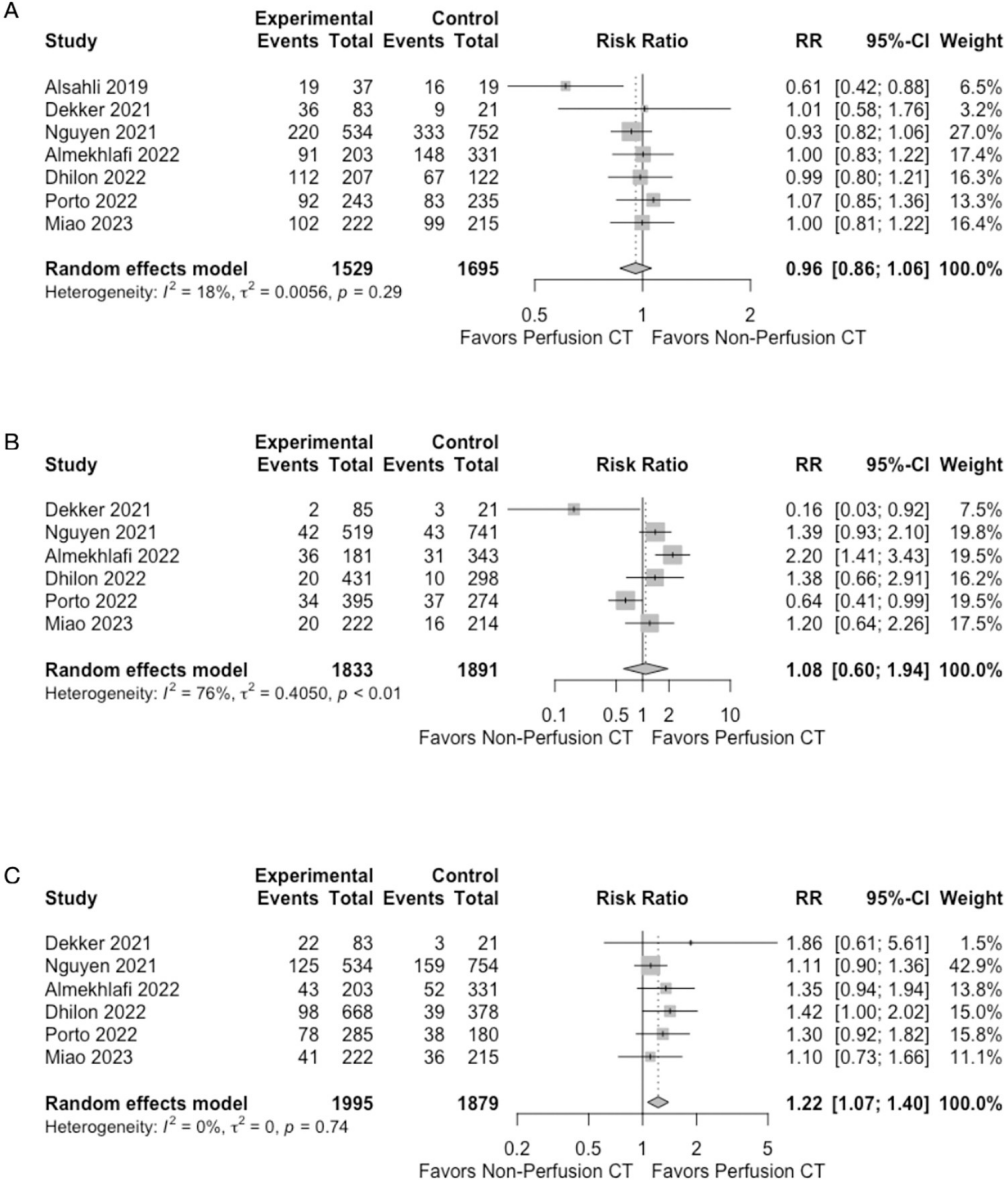
Forest plots of study outcomes: A good long term (>90 days) clinical outcomes (mRS >2), B symptomatic intracranial hemorrhage, C mortality.

We found seven observational cohorts for long term good clinical outcomes [7, 23, 25–27]. Of these, one did not have data for sICH and mortality [26] (Fig 2). The use of non-perfusion CT neuroimaging compared to perfusion CT neuroimaging was not significantly different for both long-term clinical outcomes (n = 3,224; RR: 0.96; 95% CI 0.86 to 1.06; I^2^ = 18%) and sICH (n = 3,724; RR: 1.08 95% CI 0.60 to 1.94; I^2^ = 76%). In terms of mortality however, fewer deaths were seen with perfusion CT neuroimaging (n = 3874; RR: 1.22; 95% CI 1.07 to 1.40; I^2^ = 0%) of ELVIS.

We performed sensitivity analysis by removing the study of Dhillon et al. [25] from the meta-analyses for long term clinical outcomes and mortality. The study was different form the other studies in that it looked at 6-month outcomes (vs 3-month outcomes) and looked at in-hospital mortality (vs overall mortality). Results were similar (S2 Table).

### Certainty of evidence

After assessing all the studies for each outcome using the GRADE approach, we downgraded the certainty of evidence from low, the starting point for observational studies, to very low. For all three outcomes, we found a serious limitation for risk of bias and imprecision (Table 2). Significant imprecision was found because the confidence intervals of sICH and mortality overlapped with our predetermined appreciable harm/ benefit rate, 10%. Additionally, for sICH, serious limitation for the inconsistency domain was also found (S2 Appendix).

**Table 2:** GRADE assessment.

Certainty assessment							№ of patients		Effect		Certainty	Importance
№ of studies	Study design	Risk of bias	Inconsistency	Indirectness	Imprecision	Other considerations	Basic CT Neuroimaging	Advanced CT Neuroimaging	Relative (95% CI)	Absolute (95% CI)		
Good long term clinical outcome												
6	observational studies	serious	not serious	not serious	not serious	none	672/1529 (44.0%)	573/1245 (46.0%)	RR 0.96 (0.86 to 1.06)	18 fewer per 1,000 (from 64 fewer to 28 more)	⨁◯◯◯ Very low due to risk of bias	CRITICAL
Symptomatic intracranial hemorrhage												
5	observational studies	serious	serious	not serious	serious	none	154/1833 (8.4%)	140/1891 (7.4%)	RR 1.08 (0.60 to 1.94)	6 more per 1,000 (from 30 fewer to 70 more)	⨁◯◯◯ Very low due to risk of bias, inconsistency, and imprecision	CRITICAL
Mortality												
5	observational studies	serious	not serious	not serious	serious	none	407/1995 (20.4%)	327/1879 (17.4%)	RR 1.22 (1.07 to 1.40)	38 more per 1,000 (from 12 more to 70 more)	⨁◯◯◯ Very low due to risk of bias and imprecision	CRITICAL

**CI:** confidence interval; **RR:** risk ratio

### Publication bias

Both funnel plots and Egger’s test results did not indicate significicant publication bias save for some asymmetry for the good clinical outcomes funnel and sICH funnel plots (S4 Appendix and S3 Table).

## Discussion

Our meta-analyses show that the use of non-perfusion CT neuroimaging compared to perfusion CT neuroimaging was not significantly different for both long-term clinical outcomes and sICH but favored perfusion CT neuroimaging when looking at mortality. However, the degree of certainty of these findings was very low on account of serious limitations in bias, inconsistency and imprecision domains found on the GRADE assessment.

There have been three published meta-analyses with a similar objective to ours [28–30]. However, all three studies did not exclude a considerable number (>300) duplicated patients from three cohorts included in the meta-analyses [7, 22, 23]. This may result in different results with falsely elevated precision and consistency. They also included patients who also underwent cranial MRI.

The absolute proportion of patients with good long term clinical outcomes (43.9%, 44.5.0%, 45%), sICH (8.4%, 7.4%, 7%) and mortality (20.4%, 17.4%, 14%) were similar in the non-perfusion CT neuroimaging arm, perfusion CT neuroimaging arm of our meta-analysis and the intervention arm (utilizing perfusion neuroimaging) of the DEFUSE 3 randomized trial, a study utilizing perfusion CT for all patients [4, 31].

Several reasons may account for the increased mortality seen in the non-perfusion CT neuroimaging group. The low inter-rater and intra-rater agreement seen in CT ASPECTS (Alberta Stroke Program Early CT Score) [32] may have resulted in patients with larger cores undergoing EVT treatment. The quantitative values seen with perfusion CT neuroimaging may have led patients with a higher likelihood of mortality on account of their larger cores away from intervention. A higher absolute rate of sICH in the non-perfusion CT neuroimaging group could have also contributed to higher mortality in this group. Lastly, centers utilising perfusion CT neuroimaging may be preferentially located in academic centers with greater experience and expertise resulting in less mortality. However, the results of this meta-analysis by no means imply that non-perfusion CT neuroimaging is not a feasible option for late-presenting ELVIS patients. Stringent criteria with automated perfusion imaging may result over-selection: better outcomes in treated patients but more patients left untreated to the natural history of an ELVIS [33].

Recently, three new randomized controlled trials published results on best medical management versus EVT outcomes of ELVIS patients already associated with a large are of infarction—that is, those with low ASPECTS and/or high perfusion CT estimated cores [34–36]. Early and late window patients were included in these trials. All three trials, SELECT 2, RESCUE LIMIT and ANGEL ASPECT demonstrated a statistically significant clinical benefit in the EVT arm. Consequently, the rationale behind advocating perfusion imaging to withhold treatment for strokes with extensive core infarctions appears to lack validity, as the results of these randomized trials already demonstrate that even individuals in this category are likely do better with EVT.

The rationale for the use of perfusion CT neuroimaging stems from the inclusion criteria set by DEFUSE 3, not from the actual randomization of patients between perfusion and no perfusion groups. MR CLEAN LATE, was a randomized trial that focused on late window EVT patients and randomized patients, who did not qualify for upfront EVT based on stringent perfusion CT requirements, based on non-perfusion CT neuroimaging [37]. This is similar to the large core trials, ANGEL ASPECT and TENSION, that allowed randomization based on non-perfusion CT alone in the extended time window [38, 39]. Thus, one can argue, that the evidence for non-perfusion CT neuroimaging is now at par with perfusion CT neuroimaging as both the MR CLEAN LATE and ANGEL ASPECT trials like DEFUSE 3 also had positive results. An upcoming trial, “A Randomized Trial of Imaging Selection Modalities for Stroke Thrombectomy (NO-CTP)” (NCT05230914) would give a more direct answer to our clinical question [40].

Limitations of our review process include the lack of a PRESS (Peer Review of Electronic Search Strategies) that involves the use of two information specialists instead of just one [41]. The observational nature of the studies involved in our review impart a serious degree of bias. Issues with imprecision and inconsistency also limit the certainty of our findings. Some asymmetry was also found in the funnel plot. However, the use of a funnel plot is typically recommended for reviews that are larger than ours (≥10 studies) [42]. Our search was done with an information specialist, covered major databases and references from pertinent full text literature and included ongoing studies. We feel that these methods decrease the probability of publication bias. Furthermore, it does not differentiate between single and multiphase CTA and between non-automated and automated quantitative perfusion imaging.

## Conclusion

Our meta-analyses shows that the use of non-perfusion CT neuroimaging compared to perfusion CT neuroimaging may have little or no effect for both long-term clinical outcomes and sICH but favors perfusion CT neuroimaging when looking at mortality. However, the evidence is uncertain. We await published evidence from randomized trials to provide evidence whether non-perfusion CT neuroimaging is an acceptable imaging modality for late presenting ELVIS patients.

## Supporting information

S1 Checklist PRISMA 2009 checklist (adapted for KIN 4400). (DOC)

S1 Appendix Detailed search strategies. (DOCX)

S2 Appendix Risk of bias assessment with ROBINS-I. (DOCX)

S3 Appendix Certainty of evidence—GRADE. (DOCX)

S4 Appendix Funnel plots for publication bias assessment. (DOCX)

S1 Table Table of excluded studies. (DOCX)

S2 Table Sensitivity analyses. (DOCX)

S3 Table Egger’s test. (DOCX)

## References

[1] Goyal M, Menon BK, van Zwam WH, Dippel DWJ, Mitchell PJ, Demchuk AM, et al. Endovascular thrombectomy after large-vessel ischaemic stroke: a meta-analysis of individual patient data from five randomised trials. The Lancet. 2016;387: 1723–1731. doi: 10.1016/S0140-6736(16)00163-X 26898852

[2] Powers WJ, Rabinstein AA, Ackerson T, Adeoye OM, Bambakidis NC, Becker K, et al. Guidelines for the Early Management of Patients With Acute Ischemic Stroke: 2019 Update to the 2018 Guidelines for the Early Management of Acute Ischemic Stroke: A Guideline for Healthcare Professionals From the American Heart Association/American Stroke. Stroke. 2019;50. doi: 10.1161/STR.0000000000000211 31662037

[3] Powers WJ, Rabinstein AA, Ackerson T, Adeoye OM, Bambakidis NC, Becker K, et al. 2018 Guidelines for the Early Management of Patients With Acute Ischemic Stroke: A Guideline for Healthcare Professionals From the American Heart Association/American Stroke Association. Stroke. 2018;49: e46–e99. doi: 10.1161/STR.0000000000000158 29367334

[4] Albers GW, Marks MP, Kemp S, Christensen S, Tsai JP, Ortega-Gutierrez S, et al. Thrombectomy for Stroke at 6 to 16 Hours with Selection by Perfusion Imaging. New England Journal of Medicine. 2018;378: 708–718. doi: 10.1056/NEJMoa1713973 29364767 PMC6590673

[5] Nogueira RG, Jadhav AP, Haussen DC, Bonafe A, Budzik RF, Bhuva P, et al. Thrombectomy 6 to 24 hours after stroke with a mismatch between deficit and infarct. New England Journal of Medicine. 2018;378: 11–21. doi: 10.1056/NEJMoa1706442 29129157

[6] Nguyen TN, Castonguay AC, Siegler JE, Nagel S, Lansberg MG, de Havenon A, et al. Mechanical Thrombectomy in the Late Presentation of Anterior Circulation Large Vessel Occlusion Stroke: A Guideline From the Society of Vascular and Interventional Neurology Guidelines and Practice Standards Committee. Stroke: Vascular and Interventional Neurology. 2022. doi: 10.1161/SVIN.122.000512 PMC11460660 39380893

[7] Nguyen TN, Abdalkader M, Nagel S, Qureshi MM, Ribo M, Caparros F, et al. Noncontrast Computed Tomography vs Computed Tomography Perfusion or Magnetic Resonance Imaging Selection in Late Presentation of Stroke With Large-Vessel Occlusion. JAMA Neurol. 2021. doi: 10.1001/jamaneurol.2021.4082 34747975 PMC8576630

[8] Powers WJ, Rabinstein AA, Ackerson T, Adeoye OM, Bambakidis NC, Becker K, et al. Guidelines for the Early Management of Patients With Acute Ischemic Stroke: 2019 Update to the 2018 Guidelines for the Early Management of Acute Ischemic Stroke: A Guideline for Healthcare Professionals From the American Heart Association/American Stroke. Stroke. 2019;50. doi: 10.1161/STR.0000000000000211 31662037

[9] Covidence systematic review software. Melbourne: Veritas Health Innovation; 2022.

[10] van Swieten JC, Koudstaal PJ, Visser MC, Schouten HJ, van Gijn J. Interobserver agreement for the assessment of handicap in stroke patients. Stroke. 1988;19: 604–607. doi: 10.1161/01.str.19.5.604 3363593

[11] Yaghi S, Willey JZ, Cucchiara B, Goldstein JN, Gonzales NR, Khatri P, et al. Treatment and Outcome of Hemorrhagic Transformation After Intravenous Alteplase in Acute Ischemic Stroke: A Scientific Statement for Healthcare Professionals From the American Heart Association/American Stroke Association. Stroke. 2017;48. doi: 10.1161/STR.0000000000000152 29097489

[12] Higgins JPT LTDJ. Chapter 6: Choosing effect measures and computing estimates of effect in Cochrane Handbook for Systematic Reviews of Interventions. Higgins JPT, Thomas J, Chandler J, Cumpston M, Li T, Page MJ, et al., editors. Cochrane; 2023. Available: https://training.cochrane.org/handbook/current/chapter-06

[13] Sterne JA, Hernán MA, Reeves BC, Savović J, Berkman ND, Viswanathan M, et al. ROBINS-I: a tool for assessing risk of bias in non-randomised studies of interventions. BMJ. 2016; i4919. doi: 10.1136/bmj.i4919 27733354 PMC5062054

[14] Sterne J, Higgins J, Elbers R, Reeves B, Development group for ROBINS-I. Risk Of Bias In Non-randomized Studies of Interventions (ROBINS-I): detailed guidance. 2016.

[15] Team RStudio. RStudio: Integrated Development for R. Boston; 2020.

[16] Viechtbauer W. Conducting Meta-Analyses in R with the metafor Package. J Stat Softw. 2010;36. doi: 10.18637/jss.v036.i03

[17] Veroniki AA, Jackson D, Viechtbauer W, Bender R, Bowden J, Knapp G, et al. Methods to estimate the between-study variance and its uncertainty in meta-analysis. Res Synth Methods. 2016;7: 55–79. doi: 10.1002/jrsm.1164 26332144 PMC4950030

[18] Hartung J, Knapp G. A refined method for the meta-analysis of controlled clinical trials with binary outcome. Stat Med. 2001;20: 3875–89. doi: 10.1002/sim.1009 11782040

[19] Guyatt G, Oxman AD, Akl EA, Kunz R, Vist G, Brozek J, et al. GRADE guidelines: 1. Introduction—GRADE evidence profiles and summary of findings tables. J Clin Epidemiol. 2011;64: 383–394. doi: 10.1016/j.jclinepi.2010.04.026 21195583

[20] Guyatt G, Oxman AD, Kunz R, Brozek J, Alonso-Coello P, Rind D, et al. Corrigendum to GRADE guidelines 6. Rating the quality of evidence-imprecision. J Clin Epidemiol 2011;64:1283–1293. J Clin Epidemiol. 2021;137: 265. doi: 10.1016/j.jclinepi.2021.04.014 34174652

[21] Santesso N, Glenton C, Dahm P, Garner P, Akl EA, Alper B, et al. GRADE guidelines 26: informative statements to communicate the findings of systematic reviews of interventions. J Clin Epidemiol. 2020;119: 126–135. doi: 10.1016/j.jclinepi.2019.10.014 31711912

[22] Nogueira RG, Haussen DC, Liebeskind D, Jovin TG, Gupta R, Jadhav A, et al. Stroke Imaging Selection Modality and Endovascular Therapy Outcomes in the Early and Extended Time Windows. Stroke. 2021;52: 491–497. doi: 10.1161/STROKEAHA.120.031685 33430634

[23] Almekhlafi MA, Thornton J, Casetta I, Goyal M, Nannoni S, Herlihy D, et al. Stroke imaging prior to thrombectomy in the late window: results from a pooled multicentre analysis. J Neurol Neurosurg Psychiatry. 2022;93: 468–474. doi: 10.1136/jnnp-2021-327959 35086938

[24] Miao J, Sang H, Li F, Saver JL, Lei B, Li J, et al. Effect of Imaging Selection Paradigms on Endovascular Thrombectomy Outcomes in Patients With Acute Ischemic Stroke. Stroke. 2023;54: 1569–1577. doi: 10.1161/STROKEAHA.122.042203 37165864

[25] Dhillon PS, Butt W, Podlasek A, McConachie N, Lenthall R, Nair S, et al. Perfusion Imaging for Endovascular Thrombectomy in Acute Ischemic Stroke Is Associated With Improved Functional Outcomes in the Early and Late Time Windows. Stroke. 2022;53: 2770–2778. doi: 10.1161/STROKEAHA.121.038010 35506384 PMC9389941

[26] Alsahli K, Cheung AK, Wijesuriya N, Cordato D, Zagami AS, Wenderoth JD, et al. Thrombectomy in stroke of unknown onset, wake up stroke and late presentations: Australian experience from 2 comprehensive stroke centres. Journal of Clinical Neuroscience. 2019;59: 136–140. doi: 10.1016/j.jocn.2018.10.114 30414809

[27] Dekker L, Venema E, Pirson FA V, Majoie CBLM, Emmer BJ, Jansen IGH, et al. Endovascular treatment in anterior circulation stroke beyond 6.5 hours after onset or time last seen well: results from the MR CLEAN Registry. Stroke Vasc Neurol. 2021;6: 572–580. doi: 10.1136/svn-2020-000803 33827915 PMC8717786

[28] Sequeiros JM, Rodriguez-Calienes A, Chavez-Malpartida SS, Morán-Mariños C, Alvarado-Gamarra G, Malaga M, et al. Stroke imaging modality for endovascular therapy in the extended window: systematic review and meta-analysis. J Neurointerv Surg. 2022; neurintsurg-2022-018896. doi: 10.1136/neurintsurg-2022-018896 35725306

[29] Dong Z, Deng S, Zhang J, Chen S, Ye Z, Zhang L, et al. Simplified stroke imaging selection modality for endovascular thrombectomy in the extended time window: systematic review and meta-analysis. J Neurointerv Surg. 2022; jnis-2022-019556. doi: 10.1136/jnis-2022-019556 36597953 PMC10803987

[30] Kobeissi H, Ghozy S, Adusumilli G, Bilgin C, Tolba H, Amoukhteh M, et al. CT Perfusion vs Noncontrast CT for Late Window Stroke Thrombectomy. Neurology. 2023;100: e2304–e2311. doi: 10.1212/WNL.0000000000207262 36990720 PMC10259276

[31] Powers WJ, Rabinstein AA, Ackerson T, Adeoye OM, Bambakidis NC, Becker K, et al. Guidelines for the Early Management of Patients With Acute Ischemic Stroke: 2019 Update to the 2018 Guidelines for the Early Management of Acute Ischemic Stroke: A Guideline for Healthcare Professionals From the American Heart Association/American Stroke. Stroke. 2019;50. doi: 10.1161/STR.0000000000000211 31662037

[32] Farzin B, Fahed R, Guilbert F, Poppe AY, Daneault N, Durocher AP, et al. Early CT changes in patients admitted for thrombectomy. Neurology. 2016;87: 249–256. doi: 10.1212/WNL.0000000000002860 27316243 PMC4955274

[33] Nogueira RG, Ribó M. Endovascular Treatment of Acute Stroke. Stroke. 2019;50: 2612–2618. doi: 10.1161/STROKEAHA.119.023811 31340728

[34] Yoshimura S, Sakai N, Yamagami H, Uchida K, Beppu M, Toyoda K, et al. Endovascular Therapy for Acute Stroke with a Large Ischemic Region. New England Journal of Medicine. 2022;386: 1303–1313. doi: 10.1056/NEJMoa2118191 35138767

[35] Sarraj A, Hassan AE, Abraham MG, Ortega-Gutierrez S, Kasner SE, Hussain MS, et al. Trial of Endovascular Thrombectomy for Large Ischemic Strokes. New England Journal of Medicine. 2023. doi: 10.1056/NEJMoa2214403 36762865

[36] Huo X, Ma G, Tong X, Zhang X, Pan Y, Nguyen TN, et al. Trial of Endovascular Therapy for Acute Ischemic Stroke with Large Infarct. New England Journal of Medicine. 2023. doi: 10.1056/NEJMoa2213379 36762852

[37] Olthuis SGH, Pirson FA V, Pinckaers FME, Hinsenveld WH, Nieboer D, Ceulemans A, et al. Endovascular treatment versus no endovascular treatment after 6–24 h in patients with ischaemic stroke and collateral flow on CT angiography (MR CLEAN-LATE) in the Netherlands: a multicentre, open-label, blinded-endpoint, randomised, controlled, phase 3 t. The Lancet. 2023;401: 1371–1380. doi: 10.1016/S0140-6736(23)00575-5 37003289

[38] Ren Z, Huo X, Ma G, Tong X, Kumar J, Pressman E, et al. Selection criteria for large core trials: rationale for the ANGEL-ASPECT study design. J Neurointerv Surg. 2022;14: 107–110. doi: 10.1136/neurintsurg-2021-017798 34326195 PMC8785010

[39] Bendszus M, Fiehler J, Subtil F, Bonekamp S, Aamodt AH, Fuentes B, et al. Endovascular thrombectomy for acute ischaemic stroke with established large infarct: multicentre, open-label, randomised trial. The Lancet. 2023. doi: 10.1016/S0140-6736(23)02032-9 37837989

[40] Qiu Z. A Randomized Trial of Imaging Selection Modalities for Stroke Thrombectomy (NO-CTP). 2022 [cited 16 Dec 2022]. Available: https://clinicaltrials.gov/ct2/show/NCT05230914

[41] McGowan J, Sampson M, Salzwedel DM, Cogo E, Foerster V, Lefebvre C. PRESS Peer Review of Electronic Search Strategies: 2015 Guideline Statement. J Clin Epidemiol. 2016;75: 40–46. doi: 10.1016/j.jclinepi.2016.01.021 27005575

[42] Higgins J, Thomas J, Chandler J, Cumpston M, Li T, Page M, et al. Cochrane Handbook for Systematic Reviews of Interventions version. Version 6. Cochrane; 2022 [cited 26 Nov 2022]. Available: www.training.cochrane.org/handbook

[43] Porto GBF, Chen C-J, Al Kasab S, Essibayi MA, Almallouhi E, Hubbard Z, et al. Association of Noncontrast Computed Tomography and Perfusion Modalities With Outcomes in Patients Undergoing Late-Window Stroke Thrombectomy. JAMA Netw Open. 2022;5: e2241291. doi: 10.1001/jamanetworkopen.2022.41291 36367728 PMC9652750

