# Supplementary material for: Perfusion vs non-perfusion computed tomography imaging in the late window of emergent large vessel ischemic stroke: A systematic review and meta-analysis
Source: PLoS One. 2024 Jan 2;19(1):e0294127. doi: 10.1371/journal.pone.0294127 (PMC10760723; doi:10.1371/journal.pone.0294127)
Supplement: S1 Appendix — (DOCX) [file pone.0294127.s002.docx]

**S1 Appendix: Detailed Search Strategies**

**Medline**

1 exp Thrombectomy/

2 thrombectom*.tw,kf.

3 mechanical thromb*.tw,kf.

4 mechanical clot disrupt*.tw,kf.

5 1 or 2 or 3 or 4

6 Tomography, X-Ray Computed/

7 Computed Tomography Angiography/

8 basic neuroimag*.tw,kf.

9 (non-contrast or noncontrast).tw,kf.

10 (computed tomograph* or CT).tw,kf.

11 (non-perfusion or nonperfusion).tw,kf.

12 6 or 7 or 8 or 9 or 10 or 11

13 Time-to-Treatment/ 9700

14 (late or extended or delayed).tw,kf.

15 hours.tw,kf.

16 13 or 14 or 15

17 5 and 12 and 16

18 limit 17 to yr="2015 -Current"

19 limit 18 to (case reports or comment or editorial or letter)

20 18 not 19

**Cochrane Central**

1 exp Thrombectomy/

2 thrombectom*.tw.

3 mechanical thromb*.tw.

4 mechanical clot disrupt*.tw.

5 1 or 2 or 3 or 4

6 Tomography, X-Ray Computed/

7 Computed Tomography Angiography/

8 basic neuroimag*.tw.

9 (non-contrast or noncontrast).tw.

10 (computed tomograph* or CT).tw.

11 (non-perfusion or nonperfusion).tw.

12 6 or 7 or 8 or 9 or 10 or 11

13 Time-to-Treatment/

14 (late or extended or delayed).tw.

15 hours.tw.

16 13 or 14 or 15

17 5 and 12 and 16

18 limit 17 to yr="2015 -Current"

**Embase**

1 exp percutaneous thrombectomy/

2 thrombectom*.tw,kf.

3 mechanical thromb*.tw,kf.

4 mechanical clot disrupt*.tw,kf.

5 1 or 2 or 3 or 4

6 x-ray computed tomography/

7 computed tomographic angiography/

8 basic neuroimag*.tw,kf.

9 (non-contrast or noncontrast).tw,kf.

10 (computed tomograph* or CT).tw,kf.

11 (non-perfusion or nonperfusion).tw,kf.

12 6 or 7 or 8 or 9 or 10 or 11

13 time to treatment/

14 (late or extended or delayed).tw,kf.

15 hours.tw,kf.

16 13 or 14 or 15

17 5 and 12 and 16

18 limit 17 to yr="2015 -Current"

19 limit 18 to (editorial or letter)

20 18 not 19

21 limit 20 to embase

**Scopus**

( ( TITLE-ABS-KEY ( thrombectom* ) )  OR  ( TITLE-ABS-KEY ( "mechanical thromb*" ) )  OR  ( TITLE-ABS-KEY ( "mechanical clot disrupt*" ) ) )  AND  ( ( TITLE-ABS-KEY ( "basic neuroimag*" ) )  OR  ( TITLE-ABS-KEY ( ( "non-contrast"  OR  noncontrast ) ) )  OR  ( TITLE-ABS-KEY ( ( "computed tomograph*"  OR  ct ) ) )  OR  ( TITLE-ABS-KEY ( ( "non-perfusion"  OR  nonperfusion ) ) ) )  AND  ( ( TITLE-ABS-KEY ( ( late  OR  extended  OR  delayed ) ) )  OR  ( TITLE-ABS-KEY ( hours ) ) )  AND  ( LIMIT-TO ( PUBYEAR ,  2023 )  OR  LIMIT-TO ( PUBYEAR ,  2022 )  OR  LIMIT-TO ( PUBYEAR ,  2021 )  OR  LIMIT-TO ( PUBYEAR ,  2020 )  OR  LIMIT-TO ( PUBYEAR ,  2019 )  OR  LIMIT-TO ( PUBYEAR ,  2018 )  OR  LIMIT-TO ( PUBYEAR ,  2017 )  OR  LIMIT-TO ( PUBYEAR ,  2016 )  OR  LIMIT-TO ( PUBYEAR ,  2015 ) )  AND  ( EXCLUDE ( DOCTYPE ,  "no" )  OR  EXCLUDE ( DOCTYPE ,  "le" )  OR  EXCLUDE ( DOCTYPE ,  "ed" )  OR  EXCLUDE ( DOCTYPE ,  "re" )  OR  EXCLUDE ( DOCTYPE ,  "cp" )  OR  EXCLUDE ( DOCTYPE ,  "ch" )  OR  EXCLUDE ( DOCTYPE ,  "sh" )  OR  EXCLUDE ( DOCTYPE ,  "cr" ) )
