# Supplementary material for: Perfusion vs non-perfusion computed tomography imaging in the late window of emergent large vessel ischemic stroke: A systematic review and meta-analysis
Source: PLoS One. 2024 Jan 2;19(1):e0294127. doi: 10.1371/journal.pone.0294127 (PMC10760723; doi:10.1371/journal.pone.0294127)
Supplement: S3 Appendix — (DOCX) [file pone.0294127.s004.docx]

**S3 Appendix: Certainty of Evidence—GRADE**

**Outcome: Good long term clinical outcomes**

***Risk of Bias: Serious limitation***

Some of the studies adjusted for possible confounders in their studies. However, they did not adjust for the same confounders. Thus, we used the unadjusted data for our analysis. This resulted in a serious risk of bias for all the studies using the ROBINS-I tool; consequently, we have judged the whole body of evidence to have a serious limitation.

***Inconsistency: No serious limitation***

Visual inspection shows that all the confidence intervals overlap at some point. The statistical test for heterogeneity has a p value of 0.29. Thus, we do not reject the null hypothesis. Lastly, the I^2^ is low at 18%. This may not be important heterogeneity.^5^ Thus, we will not rate down for inconsistency.

***Indirectness: No serious indirectness***

All 5 articles included:

1. Were conducted in the population stated in our research question: emergent large vessel ischemic stroke patients
2. Included the two interventions: basic CT neuroimaging and advanced CT neuroimaging
3. Measured long term clinical outcomes

The evidence gathered directly answers our research question. The outcomes of the studies were not surrogate outcomes and directly addressed the important clinical outcomes that impact the patients’ lives. Based on these, we will not downgrade for indirectness.

***Imprecision: No serious limitation***

We have more than 1000 events so our sample size should be sufficient to detect a relative risk reduction of 20%. Likewise our computed optimal information size (https://www.stat.ubc.ca/~rollin/stats/ssize/b2.html) only requires a total sample size of 200. Our sample size in this review far exceeds this at 3224. We have set our appreciable harm/ benefit rate at 25% relative risk reduction. The confidence interval of the pooled result does not cross this. Thus, we will not downgrade for imprecision.

***Publication bias: Not detected***

The funnel plot shows some asymmetry. However, it must be kept in mind that the use of a funnel plot is typically recommended for reviews that are larger than ours (at 10 studies).^6^ Our review only has 7 studies, so the funnel plot would have limited utility. Our search was done with the help of an information specialist and covered major databases (MEDLINE, EMBASE, SCOPUS, Cochrane Central Register of Controlled Trials) and references from pertinent full text literature without language limitations. We feel that our search was very comprehensive and decided not to rate down for publication bias.

***Other: No upgrade or downgrade***

The magnitude of effect found is not large enough to warrant an upgrade. A dose response effect is not applicable to our intervention. We did not find any other factors to affect the upgrade or downgrade of the effects.

***Overall certainty of evidence: VERY LOW***

The finding of a serious limitation for the risk of bias has resulted in a downgrade of our certainty of evidence to very low.

**Outcome: sICH (symptomatic intracranial hemorrhage)**

***Risk of Bias: Serious limitation***

We used the unadjusted data for our analysis. This resulted in a serious risk of bias for all the studies using the ROBINS-I tool; consequently, we have judged the whole body of evidence to have a serious limitation.

***Inconsistency: Serious limitation***

Visual inspection shows that not all the confidence intervals overlap. The statistical test for heterogeneity has a p value of <0.01. Thus, we reject the null hypothesis. Lastly, the I^2^ is high at 76%. This may be important heterogeneity.^5^ Thus, we will rate down for inconsistency.

***Indirectness: No serious limitation***

All 6 articles included:

1. Were conducted in the population stated in our research question: emergent large vessel ischemic stroke patients
2. Included the two interventions: basic CT neuroimaging and advanced CT neuroimaging
3. Measured sICH

The evidence gathered directly answers our research question. We did not have pre-planned subgroup analyses. The outcomes of the studies were not surrogate outcomes and directly addressed the important clinical outcomes that impact the patients’ lives. Based on these, we will not downgrade for indirectness.

***Imprecision: Serious limitation***

We have set our appreciable harm/ benefit rate at 10% relative risk reduction. The confidence interval of the pooled result crosses this. Thus, we will downgrade for imprecision.

***Publication bias: Not detected***

The funnel plot shows some asymmetry. However, it must be kept in mind that the use of a funnel plot is typically recommended for reviews that are larger than ours (at 10 studies).^6^ Our review only has 6 studies, so the funnel plot would have limited utility. Our search was done with the help of an information specialist and covered major databases (MEDLINE, EMBASE, SCOPUS, Cochrane Central Register of Controlled Trials) and references from pertinent full text literature without language limitations. We feel that our search was very comprehensive and decided not to rate down for publication bias.

***Other: No upgrade or downgrade***

The magnitude of effect found is not large enough to warrant an upgrade. A dose response effect is not applicable to our intervention. We did not find any other factors to affect the upgrade or downgrade of the effects.

***Overall certainty of evidence: VERY LOW***

The finding of a serious limitation for the risk of bias, inconsistency and imprecision has resulted in a downgrade of our certainty of evidence to very low.

**Outcome: Mortality**

***Risk of Bias: Serious limitation***

We used the unadjusted data for our analysis. This resulted in a serious risk of bias for all the studies using the ROBINS-I tool; consequently, we have judged the whole body of evidence to have a serious limitation.

***Inconsistency: No serious limitation***

Visual inspection shows that not all the confidence intervals. The statistical test for heterogeneity has a p value of 0.74. Thus, we do not reject the null hypothesis. Lastly, the I^2^ is high at 0%. This is not important heterogeneity.^5^ Thus, we will not rate down for inconsistency.

***Indirectness: No serious limitation***

All 6 articles included:

1. Were conducted in the population stated in our research question: emergent large vessel ischemic stroke patients
2. Included the two interventions: basic CT neuroimaging and advanced CT neuroimaging
3. Measured mortality

The evidence gathered directly answers our research question. We did not have pre-planned subgroup analyses. The outcomes of the studies were not surrogate outcomes and directly addressed the important clinical outcomes that impact the patients’ lives. Based on these, we will not downgrade for indirectness.

***Imprecision: Serious limitation***

We have 734 events. This should be sufficient to meet the threshold. We have set our appreciable harm/ benefit rate at 10% relative risk reduction. The confidence interval of the pooled result crosses this. Thus, we will downgrade for imprecision.

***Publication bias: Not detected***

The funnel plot does not show significant asymmetry. However, it must be kept in mind that the use of a funnel plot is typically recommended for reviews that are larger than ours (at 10 studies).^6^ Our review only has 6 studies, so the funnel plot would have limited utility. Our search was done with the help of an information specialist and covered major databases (MEDLINE, EMBASE, SCOPUS, Cochrane Central Register of Controlled Trials) and references from pertinent full text literature without language limitations. We feel that our search was very comprehensive and decided not to rate down for publication bias.

***Other: No upgrade or downgrade***

The magnitude of effect found is not large enough to warrant an upgrade. A dose response effect is not applicable to our intervention. We did not find any other factors to affect the upgrade or downgrade of the effects.

***Overall certainty of evidence: VERY LOW***

The finding of a serious limitation for the risk of bias and imprecision has resulted in a downgrade of our certainty of evidence to very low.
