# Supplementary material for: Perfusion vs non-perfusion computed tomography imaging in the late window of emergent large vessel ischemic stroke: A systematic review and meta-analysis
Source: PLoS One. 2024 Jan 2;19(1):e0294127. doi: 10.1371/journal.pone.0294127 (PMC10760723; doi:10.1371/journal.pone.0294127)
Supplement: S4 Appendix — (DOCX) [file pone.0294127.s005.docx]

**S4 Appendix: Funnel plots for publication bias assessment**

***Good long term clinical outcomes***

***
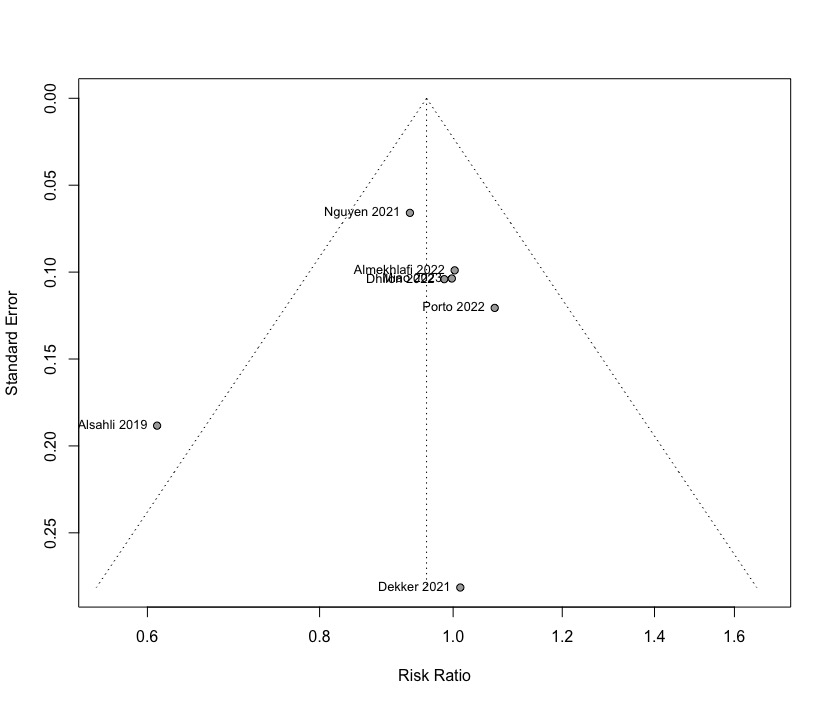
***

***Symptomatic Intracranial Hemorrhage (sICH)***


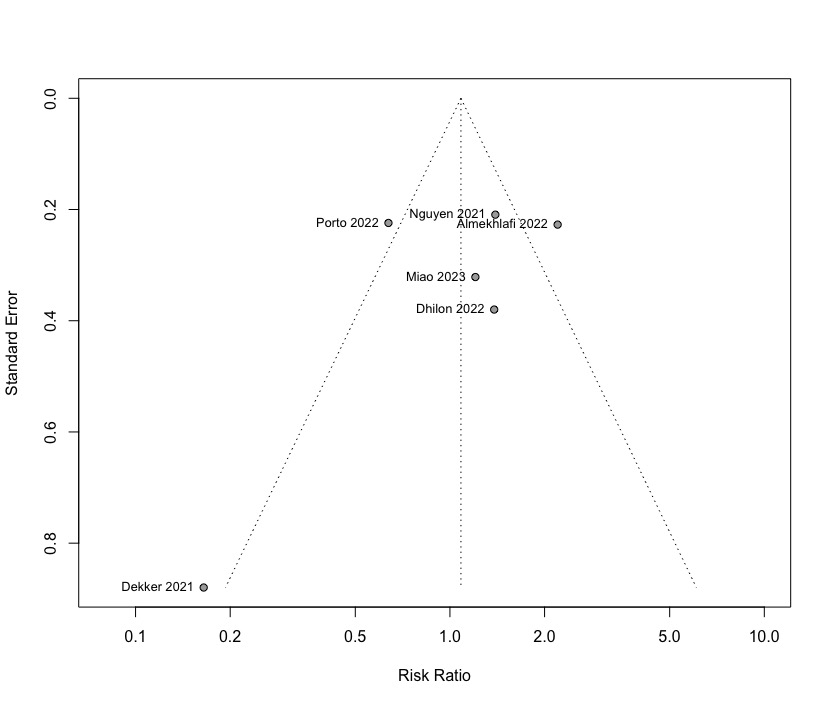


***Mortality***

**
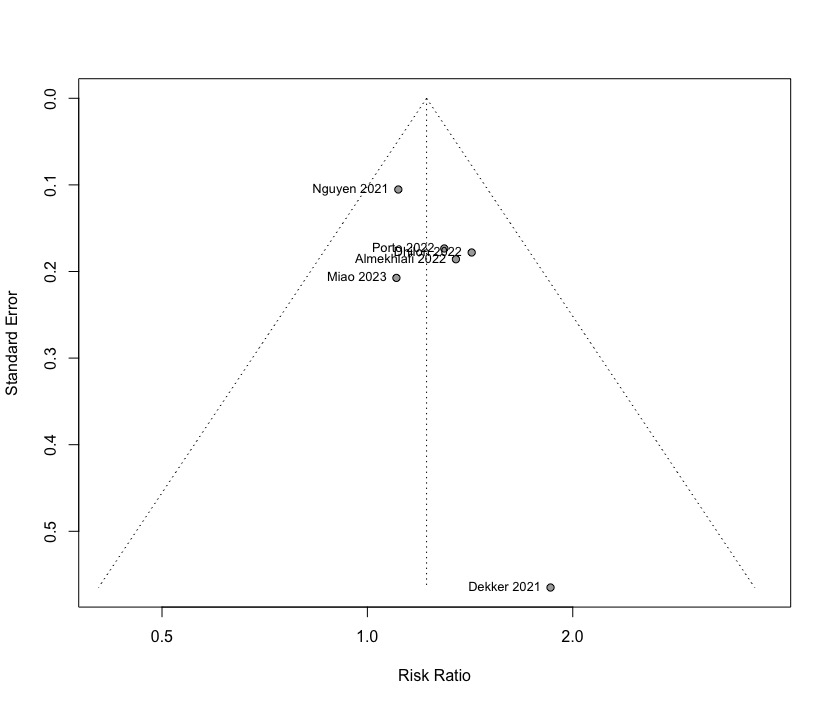
**
