# Supplementary material for: Perfusion vs non-perfusion computed tomography imaging in the late window of emergent large vessel ischemic stroke: A systematic review and meta-analysis
Source: PLoS One. 2024 Jan 2;19(1):e0294127. doi: 10.1371/journal.pone.0294127 (PMC10760723; doi:10.1371/journal.pone.0294127)
Supplement: S1 Table — (DOCX) [file pone.0294127.s006.docx]

**S1 Table: Table of Excluded Studies**

| **Primary author, Publication Year** | **Title** | **Details** |
| --- | --- | --- |
| Nogueira 2021 | Stroke Imaging Selection Modality and Endovascular Therapy Outcomes in the Early and Extended Time Windows | Significant cohort overlap with another study |
| Herzberg 2021 | Late Thrombectomy in Clinical Practice | Also used MRI for advanced imaging |
| Alexandre 2020 | May endovascular thrombectomy without CT perfusion improve clinical outcome? | Single arm only: Advanced |
| Almekhlafi 2020 | Imaging Triage of Patients with Late-Window (6-24 Hours) Acute Ischemic Stroke: A Comparative Study Using Multiphase CT Angiography versus CT Perfusion | Single arm only: Advanced |
| Beaulieu 2020 | Non-Contrast CT and CT-Angiogram for Late Window Ischemic Stroke Treatment Selection | Single arm only: Basic |
| Beckhauser 2020 | Extended Time Window Mechanical Thrombectomy for Acute Stroke in Brazil | Single arm only: Advanced |
| Bhan 2020 | Mechanical Thrombectomy for Acute Stroke: Early versus Late Time Window Outcomes | Single arm only: Advanced |
| Bhuva 2019 | Noncontrast Computed Tomography Alberta Stroke Program Early CT Score May Modify Intra-Arterial Treatment Effect in DAWN | Single arm only: Advanced |
| Bouslama 2021 | Novel selection paradigms for endovascular stroke treatment in the extended time window | Single arm only: Advanced |
| deHavenon 2019 | Results From DEFUSE 3: Good Collaterals Are Associated With Reduced Ischemic Core Growth but Not Neurologic Outcome | Single arm only: Advanced |
| DelgadoAcosta 2021 | Endovascular stroke treatment after 6-24¬†hours only needs non-contrast CT | Single arm only: Basic |
| Hendrix 2021 | Outcomes of Mechanical Thrombectomy in the Early (<6-hour) and Extended (>=6-hour) Time Window Based Solely on Noncontrast CT and CT Angiography: A Propensity Score-Matched Cohort Study | Single arm only: Basic |
| Kim 2019 | Comparison Between Perfusion- and Collateral-Based Triage for Endovascular Thrombectomy in a Late Time Window | Single arm only: Advanced |
| Kim-Tenser 2021 | CT perfusion core and ASPECT score prediction of outcomes in DEFUSE 3 | Single arm only: Advanced |
| Kraj√≠ƒçkov√° 2017 | Mechanical recanalization in ischemic anterior circulation stroke within an 8-hour time window: A real-world experience | Single arm only: Advanced |
| Krishnaiah 2022 | Yield of ASPECTS and Collateral CTA Selection for Mechanical Thrombectomy within 6-24 hours from Symptom Onset in a Hub and Spoke System | Single arm only: Basic |
| Lansberg 2019 | Association of Thrombectomy With Stroke Outcomes Among Patient Subgroups: Secondary Analyses of the DEFUSE 3 Randomized Clinical Trial | Single arm only: Advanced |
| Luo 2019 | Therapeutic effect of pre-operative tirofiban on patients with acute ischemic stroke with mechanical thrombectomy within 6-24 hours | Single arm only: Advanced |
| Motyer 2017 | Outcomes of endovascular treatment for acute large-vessel ischaemic stroke more than 6 h after symptom onset | Single arm only: Basic |
| Nannoni 2020 | Eligibility for late endovascular treatment using DAWN, DEFUSE-3, and more liberal selection criteria in a stroke center | Single arm only: Advanced |
| Nannoni 2022 | ASPECTS-based selection for late endovascular treatment: a retrospective two-site cohort study | Single arm only: Basic |
| Natera-Villalba 2021 | Mechanical thrombectomy beyond 6 hours in acute ischaemic stroke with large vessel occlusion in the carotid artery territory: experience at a tertiary hospital | Single arm only: Advanced |
| O'Connor 2020 | Predicting Clinical Outcome After Mechanical Thrombectomy: The GADIS (Gender, Age, Diabetes Mellitus History, Infarct Volume, and Sex) Score | Single arm only: Advanced |
| Santos 2019 | NCCT and CTA-based imaging protocol for endovascular treatment selection in late presenting or wake-up strokes | Single arm only: Basic |
| Siegler 2020 | Noncontrast CT versus Perfusion-Based Core Estimation in Large Vessel Occlusion: The Blood Pressure after Endovascular Stroke Therapy Study | Single arm only: Advanced |
| Virtanen 2022 | Thrombectomy in acute ischemic stroke in the extended time window: Real-life experience in a high-volume center | Single arm only: Advanced |
| Yang 2019 | Complete Recanalization May Exert the Most Important Effect on Outcomes of Endovascular Treatment in Acute Ischemic Stroke with Small Infarct Core Beyond 6 Hours | Single arm only: Basic |
