# Supplementary material for: Perfusion vs non-perfusion computed tomography imaging in the late window of emergent large vessel ischemic stroke: A systematic review and meta-analysis
Source: PLoS One. 2024 Jan 2;19(1):e0294127. doi: 10.1371/journal.pone.0294127 (PMC10760723; doi:10.1371/journal.pone.0294127)
Supplement: S2 Table — (DOCX) [file pone.0294127.s007.docx]

**S2 Table. Sensitivity Analyses**

| **Sensitivity Analyses** | **RR** | **95% Confidence Interval** | **Heterogeneity** | **Comparison with main result** |
| --- | --- | --- | --- | --- |
| Long term clinical outcomes without Dhillon et al. | 0.95 | 0.83 to 1.08 | 1^2^ = 31% | Similar |
| Mortality without Dhillon et al. | 1.19 | 1.03 to 1.38 | 1^2^ = 0% | Similar |
