# Supplementary material for: Perfusion vs non-perfusion computed tomography imaging in the late window of emergent large vessel ischemic stroke: A systematic review and meta-analysis
Source: PLoS One. 2024 Jan 2;19(1):e0294127. doi: 10.1371/journal.pone.0294127 (PMC10760723; doi:10.1371/journal.pone.0294127)
Supplement: S3 Table — (DOCX) [file pone.0294127.s008.docx]

**S3 Table. Egger’s Test**

| **Outcomes** | **Intercept** | **95% CI** | **t** | **p-value** |
| --- | --- | --- | --- | --- |
| Long term clinical outcomes | -0.6331717 | (-3.019855, 1.753512) | -0.5199657 | 0.6252799 |
| sICH | -2.1389 | (-6.909658, 2.631858) | -0.8787214 | 0.4291703 |
| Mortality | 1.23985 | (-0.008491447, 2.488191) | 1.946632 | 0.1234335 |
